# Threshold effect and sex characteristics of the relationship between chronic inflammation and BMI

**DOI:** 10.1186/s12902-023-01396-1

**Published:** 2023-08-16

**Authors:** Su Liqiang, Li Fang-Hui, Quan Minghui, Chen Haichun

**Affiliations:** 1 Physical Education College, Jiangxi Normal University, Nanchang, 330022 Jiangxi China; 2 School of Sport Sciences, Nanjing Normal University, Nanjing, 210023 Jiangsu China; 3 School of Kinesiology, Shanghai University of Sport, Shanghai, 200438 China; 4 Key Laboratory of Kinesiological Evaluation General Administration of Sport of China, School of Physical Education and Sport Science, Fujian Normal University, Fuzhou, 350108 Fujian China

**Keywords:** Chronic inflammatory, Obesity, Sex, BMI, hs-CRP

## Abstract

Chronic inflammation is an important pathway for obesity to harm health, the aggravation of chronic inflammation occurs without clinical symptoms. BMI is closely related to chronic inflammation, and it is a predictive factor of chronic inflammation, but the following questions remain unanswered: Are the effects of chronic inflammation on different BMI intervals consistent? Are the effects of BMI on chronic inflammation consistent between male and female? This study aimed to explore the threshold effect, and sex characteristics of the relationship between chronic inflammation and BMI. Methods: People with normal weight, overweight, and obesity were selected as subjects for cross-sectional study. BMI, hs-CRP, adiponectin and irisin was tested. Multiple regression analysis and generalized additive models were used to examine the association between hs-CRP and BMI. Results: 119 adults were recruited (normal weight: n = 30, 28.1 ± 7.65 years, BMI: 22.04 ± 1.55; overweight: n = 29, 27.45 ± 7.47 years, BMI: 26.11 ± 1.22; and obesity: n = 60, 28.82 ± 6.05 years, BMI: 33.68 ± 3.57). After adjusting for age and sex, BMI was found to be positively associated with the chronic inflammatory marker hs-CRP (*β* = 0.45; *P* < 0.001), which had a threshold effect on hs-CRP. Positive correlation with hs-CRP was observed for BMI of > 24.6 (*β* = 0.54; *P* < 0.001) but not for BMI of 18.5–25.4 (*β* = −0.02; *P* > 0.05). The pro-inflammatory effect caused by BMI increase in female (*β* = 0.56; *P* < 0.001) was higher than that in male (*β* = 0.38; *P* < 0.001). When BMI was greater than 33, a positive correlation with hs-CRP was observed in female (*β* = 0.97; *P* < 0.001) but not in male (*β* = 0.14; *P* > 0.05).

**Conclusions**: BMI has a threshold effect on chronic inflammation, BMI greater than 24.3 is positively correlated with hs-CRP. BMI in 18.5–24.3 is not correlated with hs-CRP. Furthermore, when the BMI greater than 33, hs-CRP is not positively correlated with BMI in male, whereas the pro-inflammatory effect of BMI increase becomes greater in female.

**Highlights:**

• BMI has a threshold effect on chronic inflammation. BMI in 18.5–24.3 is not correlated with chronic inflammation, and BMI greater than 24.3 is positively correlated with chronic inflammation.

• The pro-inflammatory effect caused by BMI increase in female is higher than that in male. In particular, when the BMI is greater than 33, chronic inflammation is not positively correlated with BMI in male, whereas the pro-inflammatory effect of BMI increase becomes greater in female.

**Supplementary Information:**

The online version contains supplementary material available at 10.1186/s12902-023-01396-1.

## Introduction

The incidence of obesity, obesity-related metabolic diseases, tumors, and other diseases has increased sharply worldwide [1–4]. These diseases bring huge burden to families and society [5–7]. Obesity endangers health through chronic inflammation [8]. Garvin et al. [9] showed that the increase in chronic inflammation markers IL-6 and CRP is related to obesity. Ying W et al. [10] found that chronic inflammation associated with obesity. The development of chronic inflammation in people with obesity often lacks obvious clinical symptoms. Therefore, understanding the relationship between BMI increase and aggravated chronic inflammation plays an important role in preventing the occurrence of obesity and obesity-related diseases.

In general, chronic inflammation is evaluated on the basis of the intermediate products or outcome products of the inflammatory reaction process[11]. TNF-α is primarily produced by adipocytes and macrophages in adipocytes, which can reveal chronic inflammation by reflecting the activation of these cells [12, 13]. hs-CRP is produced in the liver stimulated by many inflammatory mediators [11], which reflects a wide and diffuse range of inflammation. In the study of obesity and chronic inflammation, some scholars used TNF-α [14], CRP [15], and hs-CRP to evaluate chronic inflammation [16, 17]. TNF-α and hs-CRP are reliable markers for evaluating obesity-related chronic inflammation [18, 19].

BMI increase is accompanied by the aggravation of chronic inflammation [20, 21]. Scholars included people with normal weight, overweight, and obesity as subjects and found that hs-CRP increased with BMI [22]. BMI is considered to be a risk factor for predicting chronic inflammation. Many studies have shown that chronic inflammation can show different characteristics in different sex. Chang et al. found significant differences in hs-CRP among different sex[23], which may be related to sex differences in chromosomes, sex hormones, and other factors[24]. Therefore, sex factors should be paid attention to when exploring chronic inflammation. Obesity-related chronic inflammation is sex specific[25]. In addition, chronic inflammation is associated with obesity, and this association might be modified by sex[26]. Although the relationship between chronic inflammation and BMI has been widely studied, the following questions remain unanswered: Are the effects of chronic inflammation on different BMI intervals consistent? Are the effects of BMI on chronic inflammation consistent between male and female? Solving these problems may play an important role in the targeted prevention and treatment of chronic inflammation aggravation caused by BMI increase.

People with normal weight, overweight, and obesity were selected as subjects for this cross-sectional study. The influence of BMI on chronic inflammation was analyzed on the basis of testing age, height, weight, and chronic inflammation markers. The threshold effect of BMI on chronic inflammation was determined by adjusting age and sex through regression analysis.

## Materials and methods

This study is a prospective cross-sectional study, and follow the Strengthening the Reporting of Observational Studies in Epidemiology (STROBE) statement (Supplementary Table 1).

### Participants and recruitment

The investigation proceeded by means of a successive sampling study. Volunteers from the Yangzhou Lipan weight loss training camp were recruited from May 2020 to August 2020. The inclusion criteria were as follows: age ≥ 18 years old, BMI ≥ 18.5, regardless of sex. The exclusion criteria were as follows: those with inflammatory events, infectious diseases, long-term medication, hypertension, diabetes, professional physical training, and sports contraindications. During training, the health records of the volunteers were obtained. This study was ethically approved by Fujian Normal University Ethics Committee with clinical trial registration number ChiCTR2200058959. All participants signed an informed consent form for this study.

### Outcome measures

Key outcomes included basic information, irisin, adiponectin, and hs-CRP. Basic information included name, sex, age, height, weight, physical examination, disease, medication, and sports injury. The methods for testing key outcome indicators are based on literature [27, 28].

#### Body composition assessment

Height (0.1 cm) was measured using an instrument, and weight was assessed using a body composition analyzer. During the test, the participants wore slippers, hats, and single clothes. Test conditions were maintained before and after the repeated test. Body weight was presented in kilograms (kg). BMI was calculated as weight (kg) ÷ [height (m)]^2^. The Chinese BMI criteria[29, 30] were as follows: normal weight: 18.5 ≤ BMI < 24, overweight: 24 ≤ BMI < 28, obesity: BMI ≥ 28.

#### Testing of blood chronic inflammatory factors such as hs-CRP, adiponectin, and irisin

Blood indexes were measured as follows. Blood was extracted from the volunteers on an empty stomach from 7:00 to 8:00 in the morning of the second day of training. They fasted after 22:00 in the previous day. Five milliliters of blood was collected from the elbow vein of negative pressure blood collection vessel containing a separating gel coagulant. After the sample was allowed to stand at room temperature for 30 min, it was centrifuged at 3000 rpm for 5 min. The first 1 mL serum was removed and placed into a 1.5 mL antifreeze centrifuge tube. Liquid nitrogen preservation was performed to test irisin and adiponectin. In addition, relevant ELISA kits were purchased from Wuhan Yilairuite Biotechnology Co., Ltd. The second serum was kept in the blood collection vessel at 4 ℃, and hs-CRP was tested in the Nanjing Aidikang medical laboratory center.

#### Statistical analysis

All continuous variables were expressed as means ± standard deviations, and categorical data were presented as frequencies and percentages, the results of regression analysis are described as regression coefficient β (95% confidence interval) *P* value. All tests were evaluated for normality using Shapiro-Wilk statistics. All data were included in the final analyses.

The Chi-squared test was used to compare the differences in categorical variables. One-way ANOVA was used to compare the differences between different groups of continuous variables and normal distribution, and the Kruskal–Wallis rank sum test was used for those with non-normal distribution. Smooth curve fitting was performed according to the generalized additive model. The linear relationship was described by a univariate linear regression model. The threshold effect analysis was used to describe the linear/nonlinear relationship between the concentration of hs-CRP and BMI. Threshold effect analysis identifies nonlinear relationship key point (*K*) exploring the relationship hs-CRP and BMI, threshold effect model was a two-piece-wise regression model (< *K* effect 1 and > *K* effect 2) [31]. The interfering variables are adjusted to reduce bias when conducting regression models. Using sex as subgroups, analyze sex characteristics of BMI affecting hs-CRP through Smooth curve fitting and threshold effect analysis. A multiple regression analysis model with covariates was powered using the predictor variables established in each of the multivariable model and performed using G-power3.1.9.2 [32, 33]. *R*^2^ was used to measure the effect quantity [34]. According to Cohen (1998) [35], when *R*^2^ is 0.13 in regression analysis, it can achieve medium test efficiency. The effect size *f*^*2*^ of the regression model was calculated as 0.15 with *R*^2^ = 0.13. α Error was set to 0.05; power (1-*β* Error) was set to 0.9, and the estimated target sample size was n = 108 participants. Use regression imputation to fill in missing data. The data were processed and analyzed by statistical packages R (R foundation, version 3.1.2) and empower (R). *P* < 0.05 and *P* < 0.01 indicate that the difference is statistically significant.

## Results

### Study population characteristics

**Fig. 1:**
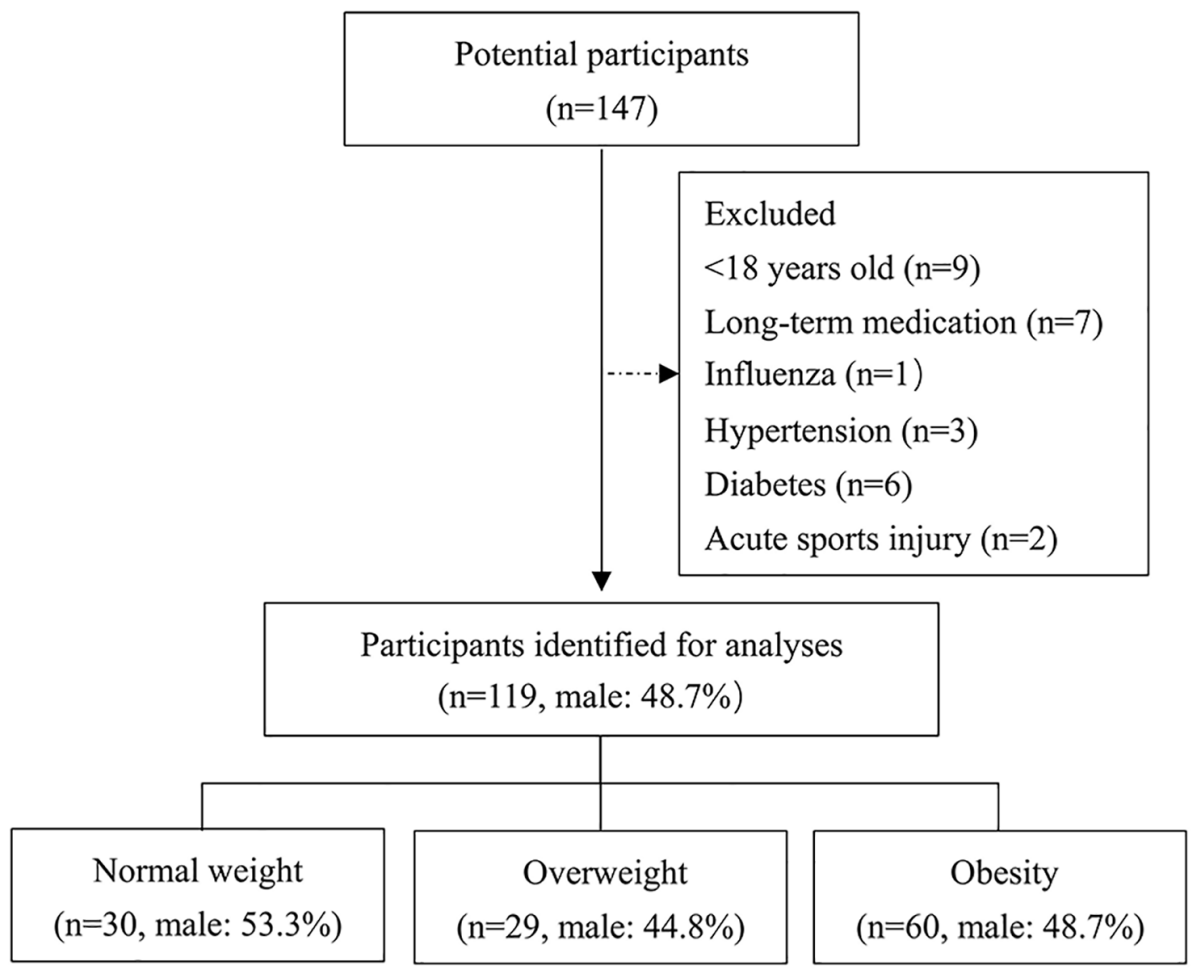
Participant flow diagram

119 adults (28.30 ± 6.80 years; male, n = 58; female, n = 61) were included in the final analysis (Fig. 1). Differences in participant characteristics across the three study cohorts are presented in Tables 1 and 2. No significant difference in age, height, irisin, and adiponectin was observed among the three groups (*P* > 0.05). Significant differences in body weight, BMI, and chronic inflammation marker hs-CRP were found among the three groups (*P* < 0.01). Significant differences in body weight and height were found between male and female (*P* < 0.01). Furthermore, no significant difference in age, BMI, irisin, adiponectin, and hs-CRP was observed between male and female (*P* > 0.05).

**Table 1:** Characteristics of participants with different BMI

	Normal weight N = 30	Overweight N = 29	Obesity N = 60	*P*
Age (years)	28.10 ± 7.65	27.45 ± 7.47	28.82 ± 6.05	0.665
Height (m)	167.27 ± 7.60	168.74 ± 7.75	170.53 ± 8.68	0.193
Weight (kg)	61.70 ± 6.17	74.52 ± 8.10	98.78 ± 18.32	< 0.001
BMI (kg/m^2^)	22.04 ± 1.55	26.11 ± 1.22	33.68 ± 3.57	< 0.001
Irisin (ng/mL)	108.62 ± 36.53	105.14 ± 30.10	118.51 ± 51.80	0.339
Adiponectin (µg/mL)	12.89 ± 7.16	10.62 ± 4.69	8.65 ± 4.93	0.003
hs-CRP (mg/L)	1.59 ± 0.66	2.12 ± 0.98	6.49 ± 4.64	< 0.001

**Table 2:** Characteristics of male and female participants

	Male n = 58	Female n = 61	*P*
Age (years)	27.40 ± 7.85	29.16 ± 5.54	0.157
Height (m)	175.81 ± 5.37	163.06 ± 5.08	< 0.001
Weight (kg)	91.58 ± 23.60	75.86 ± 15.42	< 0.001
BMI (kg/m^2^)	29.29 ± 6.35	28.52 ± 5.09	0.463
Irisin (ng/mL)	118.51 ± 50.60	107.29 ± 35.80	0.164
Adiponectin (µg/mL)	10.73 ± 6.48	9.70 ± 4.96	0.327
hs-CRP (mg/L)	3.94 ± 3.77	4.43 ± 4.36	0.517

Unit description: height, cm; weight, kg; adiponectin, µg/mL; irisin, ng/mL; hs-CRP, mg/L

### Relationship between BMI and chronic inflammation

**Table 3:** Relationship between BMI and hs-CRP

	Linear regression model	Threshold effect model		
		*K*	< *K* effect 1	> *K* effect 2
Model 1	0.44 (0.33, 0.54) < 0.0001	24.3	−0.01 (− 0.53, 0.52) 0.9834	0.51 (0.38, 0.64) < 0.0001
Model 2	0.44 (0.34, 0.54) < 0.0001	24.3	−0.11 (− 0.64, 0.42) 0.6927	0.53 (0.40, 0.66) < 0.0001
Model 3	0.44 (0.34, 0.55) < 0.0001	24.6	0.07 (− 0.43, 0.56) 0.7906	0.51 (0.38, 0.64) < 0.0001
Model 4	0.45 (0.35, 0.55) < 0.0001	24.6	−0.02 (− 0.52, 0.47) 0.9248	0.54 (0.40, 0.67) < 0.0001

Model 1, unadjusted model; Model 2, adjusted for sex; Model 3, adjusted for age; Model 4, adjusted for age and sex; *K*, nonlinear relationship key point

Using hs-CRP as the dependent variable and BMI as the independent variable, regression analysis showed that BMI was positively correlated with hs-CRP (*β* = 0.44; *P* < 0.01). After adjustment for sex or age or sex and age, BMI was found to be positively correlated with hs-CRP (*β* = 0.44, *P* < 0.01; *β* = 0.44, *P* < 0.01; *β* = 0.45, *P* < 0.01; Table 3).

The generalized additive models were then used for threshold analysis, and the results showed the threshold effect of BMI on hs-CRP with a BMI influence point at 24.3. Therefore, hs-CRP was positively correlated with BMI of > 24.3 (*β* = 0.51, *P* < 0.001) but not with BMI of 18.5–24.3 (*β* = −0.01; *P* > 0.05). Moreover, after adjustment for sex or age or sex and age, BMI had a threshold effect on hs-CRP with BMI influence point at 24.3, 24.6, and 24.6 (Table 3).

### Curve fitting of BMI on chronic inflammation

**Fig. 2:**
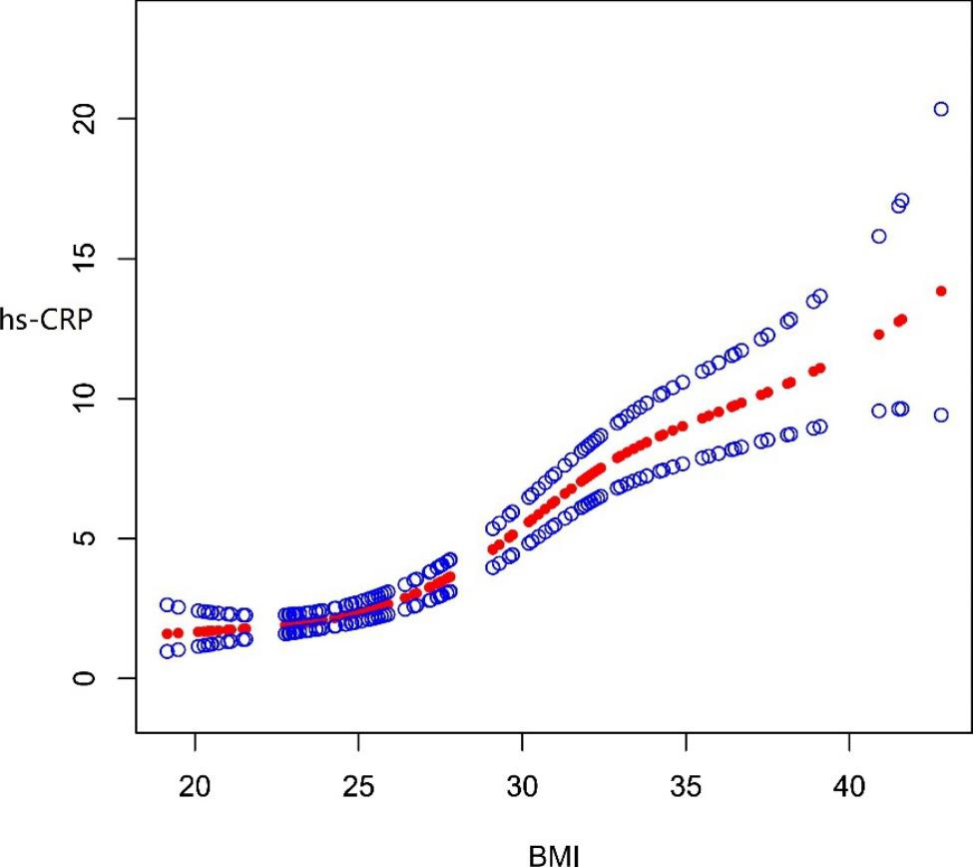
Curve fitting relationship between BMI and hs-CRP Adjusted for age and sex

**Fig. 3:**
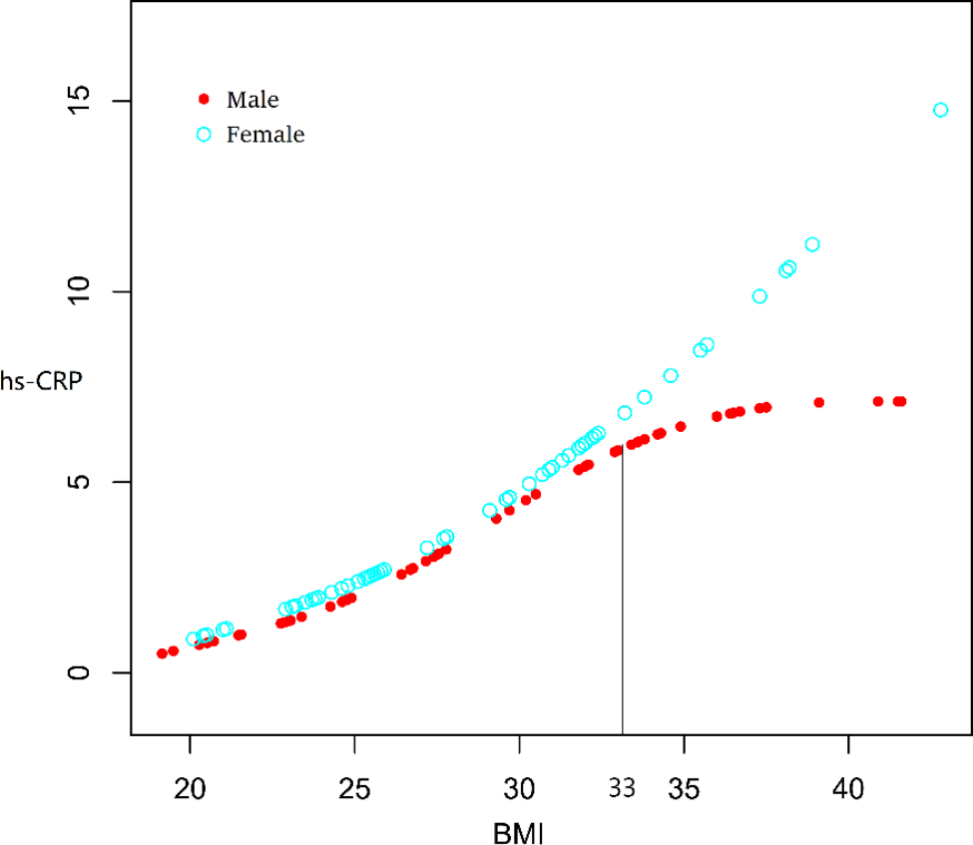
Curve fitting relationship between BMI and hs-CRP in male and female Adjusted for age and stratified for sex

Using hs-CRP as the dependent variable, BMI as the independent variable, and age and sex as the adjustment variables, curve fitting showed that BMI had a curve relationship with hs-CRP (Fig. 2). By contrast, using hs-CRP as the dependent variable, BMI as the independent variable, and age as the adjustment variable, curve fitting showed that BMI had a different curve relationship with hs-CRP in male and female when BMI was more than 33 (Fig. 3).

**Table 4:** Relationship between BMI and hs-CRP in male and female

	Outcome	Linear regression model	Threshold effect model		
			*K*	< *K* effect 1	> *K* effect 2
Model 1	Male	0.36 (0.23, 0.48) < 0.0001	33	0.46 (0.27, 0.65) < 0.0001	0.08 (-0.33, 0.49) 0.7001
	Female	0.56 (0.40, 0.73) < 0.0001	33	0.42 (0.19, 0.65) 0.0006	0.97 (0.46, 1.49) 0.0005
Model 2	Male	0.38 (0.25, 0.51) < 0.0001	33	0.47 (0.28, 0.65) < 0.0001	0.14 (-0.28, 0.56) 0.5290
	Female	0.56 (0.39, 0.73) < 0.0001	33	0.42 (0.19, 0.66) 0.0007	0.97 (0.45, 1.50) 0.0005

Model 1: unadjusted model, Model 2: adjusted for age, *K*, nonlinear relationship key point

Using hs-CRP as the dependent variable and BMI as the independent variable, regression analysis showed that BMI was positively correlated with hs-CRP (*β* = 0.36, *P* < 0.01 in male and *β* = 0.56, *P* < 0.01 in female). After adjustment for age, BMI was found to be positively correlated with hs-CRP (*β* = 0.38, *P* < 0.01 in male and *β* = 0.56, *P* < 0.01 in female; Table 4). The generalized additive models were then used for threshold analysis, and the *K* value was set to 33 (Fig. 3). The results showed that hs-CRP was not correlated with BMI > 33 (*β* = 0.08, *P* > 0.05 in male), but hs-CRP was positively correlated with BMI > 33 (*β* = 0.97, *P* < 0.01 in female). After adjustment for age, hs-CRP was not correlated with BMI > 33 (*β* = 0.14, *P* > 0.05 in male), but hs-CRP was positively correlated with BMI > 33 (*β* = 0.97, *P* < 0.01 in female; Table 4).

## Discussion

This study found that BMI is an independent risk factor for chronic inflammation. However, great differences in body health level occur in different BMI intervals [36, 37]. Wedell-Neergaard AS[38] found that BMI is not related to chronic inflammation, possibly because the BMI of the included population is concentrated between 22 and 26. Samara A[39] found that BMI is significantly correlated with chronic inflammation, and the BMI of the included population is between 19 and 33. It can be seen that the relationship between BMI and chronic inflammation varies among different BMI intervals. This study found a segmentation effect between the change of chronic inflammation marker hs-CRP and BMI. Chronic inflammation was not related to BMI ranging from 18.5 to 24.3, but it significantly increased in individuals with BMI of > 24.3. The accumulation of chronic inflammation occurs in the overweight stage. Therefore, attention must be paid to the overweight stage to prevent the aggravation of chronic inflammation. In previous reports, the blood CRP content in the overweight group was higher than that in the standard study group[40], and the abnormal rate of CRP content in the blood of 33 overweight volunteers was higher than that in the normal weight group [41]. These results showed that chronic inflammation has begun to gradually appear in the overweight stage, which is consistent with the present results. The overweight stage is the beginning of the accumulation and intensification of chronic inflammation and the beginning of excess energy. Excess energy may initially induce chronic inflammation, prompt the hypertrophy of adipocytes, lead to the relative hypoxia of intracellular organelles, increase the oxidative stress of the endoplasmic reticulum, and cause the release of proinflammatory factors into the blood [42]. Excess nutrition can cause lipid metabolism disorder and excessive fatty acid accumulation, which can promote the secretion of inflammatory factors by immune cells and cause slow inflammation. Combined with the abovementioned findings, the present results showed that chronic inflammation is aggravated when BMI is > 24.3. Therefore, controlling body weight in the overweight stage is crucial to prevent the aggravation of chronic inflammation.

Significant differences in the immune system are observed between adult male and female[43], which makes the characteristics of chronic inflammation different between the two sex[24, 26, 44]. This study found that the pro-inflammatory effect of BMI increase was higher in female than in male. Moreover, when BMI > 33, the pro-inflammatory effect of BMI increase is greater in female, and no aggravating trend of chronic inflammation was observed in male. Therefore, the mechanism of chronic inflammation caused by BMI increase in different sex may be different. BMI increase is a process of energy accumulation, and the accumulated excess energy can be stored in adipose tissue. Adipose tissue is mostly composed of adipocytes, and adipose tissue mass is determined on the basis of the number and size of adipocytes. Female tends to show an increase in fat mass based on an increase in the number and size of adipocytes, whereas men mostly show an increase in the size of adipocytes, predisposing female to systemic inflammation[45]. Therefore, the different anti-inflammatory characteristics of adipose tissue in different sex may cause the different anti-inflammatory efficacy of BMI increase in different sex.

Some studies found that the expression of adiponectin and irisin may be different between male and female, and they both have anti-inflammatory effects[46–50]. This study showed no significant difference in the concentration of irisin and adiponectin between sex, and the average concentration of female was lower than that of male. Therefore, the stronger pro-inflammatory effect of BMI increase in female may be related to the decreased protection of anti-inflammatory effect in adiponectin and irisin. Ter Horst et al.[51] have shown that females usually show lower adiponectin concentrations, whereas the decrease of serum adiponectin concentration is related to chronic inflammation [52]. In the healthy population, the concentration of serum irisin in female is lower than that in male[53]. Irisin alleviates inflammation through inhibition of NLRP3 inflammasome and NF-κB signaling [47, 54–56]. Insulin also plays an important role in regulating chronic inflammation. Wiebe N et al. [57] showed that when BMI is greater than 35, female’s fasting insulin levels are lower than male’s, and insulin has a certain anti-inflammatory effect, which may be the mechanism by which female exhibit greater pro-inflammatory effects at larger BMI.

Although adipose tissue, adiponectin, irisin, and insulin partially explain that the pro-inflammatory effect of BMI increase in female is higher than that in male, there is no direct proof that these indicators can initiate the inflammatory cascade in this study. systematic and large-scale investigations are necessary to determine the factors influencing the response of female with obesity to inflammation.

This study also has some limitations. The sample size is small, which only meets the medium efficiency of statistics. People with BMI less than 18.5 were not included, and the change law of chronic inflammation was not reflected in this range. Moreover, there are still some uncontrollable interfering factors in exploring the relationship between chronic inflammatory characteristics and BMI. Although our multiple regression analysis was adjusted, some residual confounding factors cannot be ruled out. In particular, not all potential confounders were considered in the adjustment, such as psychosocial factors and nutritional status, which could affect chronic inflammation.

## Conclusions

Within a certain range, BMI is an important risk factor for chronic inflammation, BMI has a threshold effect on chronic inflammation, BMI greater than 24.3 is positively correlated with hs-CRP. BMI in 18.5–24.3 is not correlated with hs-CRP. Moreover, when BMI is greater than 33, there may be sex differences in the relationship between BMI and chronic inflammation.

### Electronic supplementary material

Below is the link to the electronic supplementary material.

Supplementary Material 1

## Data Availability

The datasets used in the analyses described in this study are available from the corresponding author on reasonable request.
