# Supplementary material for: Threshold effect and sex characteristics of the relationship between chronic inflammation and BMI
Source: BMC Endocr Disord. 2023 Aug 16;23:175. doi: 10.1186/s12902-023-01396-1 (PMC10428651; doi:10.1186/s12902-023-01396-1)
Supplement: Supplementary file 1 — Supplementary Material 1 [file 12902_2023_1396_MOESM1_ESM.doc]

Supplementary Table 1 STROBE Statement—checklist of items

|  | Item No | Recommendation | Reflected in the manuscript |
| --- | --- | --- | --- |
| **Title and abstract** | 1 | (*a*) Indicate the study’s design with a commonly used term in the title or the abstract | Line : 19-20 |
|  |  | (*b*) Provide in the abstract an informative and balanced summary of what was done and what was found | Line : 19-20 |
| Introduction | | |  |
| Background/rationale | 2 | Explain the scientific background and rationale for the investigation being reported | Line : 44-51  Line : 66-71 |
| Objectives | 3 | State specific objectives, including any prespecified hypotheses | Line : 72-77 |
| Methods | | |  |
| Study design | 4 | Present key elements of study design early in the paper | Line : 77-78  Line : 85-87 |
| Setting | 5 | Describe the setting, locations, and relevant dates, including periods of recruitment, exposure, follow-up, and data collection | Line : 80-82 |
| Participants | 6 | *Cross-sectional study*—Give the eligibility criteria, and the sources and methods of selection of participants | Line : 82-85 |
| Variables | 7 | Clearly define all outcomes, exposures, predictors, potential confounders, and effect modifiers. Give diagnostic criteria, if applicable | Line : 88-107 |
| Data sources/ measurement | 8 | For each variable of interest, give sources of data and details of methods of assessment (measurement). Describe comparability of assessment methods if there is more than one group | Line : 88-107 |
| Bias | 9 | Describe any efforts to address potential sources of bias | Line : 121 |
| Study size | 10 | Explain how the study size was arrived at | Line : 123-128 |
| Quantitative variables | 11 | Explain how quantitative variables were handled in the analyses. If applicable, describe which groupings were chosen and why | Line : 96-97  Line : 122-123 |
| Statistical methods | 12 | (*a*) Describe all statistical methods, including those used to control for confounding | Line : 109-112 |
|  |  | (*b*) Describe any methods used to examine subgroups and interactions | Line : 122-123 |
|  |  | (*c*) Explain how missing data were addressed | Line : 128-129 |
|  |  | (*d*) *Cross-sectional study*—If applicable, describe analytical methods taking account of sampling strategy | Line : 80-81 |
|  |  | (*e*) Describe any sensitivity analyses | Line : 116-123 |

Continued on next page

| Results | | | |
| --- | --- | --- | --- |
| Participants | 13 | (a) Report numbers of individuals at each stage of study—eg numbers potentially eligible, examined for eligibility, confirmed eligible, included in the study, completing follow-up, and analysed | Line : 141 |
|  |  | (b) Give reasons for non-participation at each stage | Line : 141 |
|  |  | (c) Consider use of a flow diagram | Line : 141 |
| Descriptive data | 14 | Give characteristics of study participants (eg demographic, clinical, social) and information on exposures and potential confounders | Line : 144-147 |
| Outcome data | 15 | Report numbers of outcome events or summary measures | Line : 134-140 |
| Main results | 16 | (*a*) Give unadjusted estimates and, if applicable, confounder-adjusted estimates and their precision (eg, 95% confidence interval). Make clear which confounders were adjusted for and why they were included | Line : 154-187 |
|  |  | (*b*) Report category boundaries when continuous variables were categorized | Line : 154-187 |
|  |  | (*c*) If relevant, consider translating estimates of relative risk into absolute risk for a meaningful time period | Line : 154-187 |
| Other analyses | 17 | Report other analyses done—eg analyses of subgroups and interactions, and sensitivity analyses | Line : 171-187 |
| Discussion | | |  |
| Key results | 18 | Summarise key results with reference to study objectives | Line : 195-199  Line : 215-218 |
| Limitations | 19 | Discuss limitations of the study, taking into account sources of potential bias or imprecision. Discuss both direction and magnitude of any potential bias | Line : 243-245 |
| Interpretation | 20 | Give a cautious overall interpretation of results considering objectives, limitations, multiplicity of analyses, results from similar studies, and other relevant evidence | Line : 245-249 |
| Generalisability | 21 | Discuss the generalisability (external validity) of the study results | Line : 210-212  Line : 223-225 |
| Other information | | |  |
| Funding | 22 | Give the source of funding and the role of the funders for the present study and, if applicable, for the original study on which the present article is based | Line : 266-268 |

STROBE: The Strengthening the Reporting of Observational Studies in Epidemiology.
